# Unravelling the Functional Biomechanics of Dental Features and Tooth Wear

**DOI:** 10.1371/journal.pone.0069990

**Published:** 2013-07-23

**Authors:** Stefano Benazzi, Huynh Nhu Nguyen, Ottmar Kullmer, Jean-Jacques Hublin

**Affiliations:** 1 Department of Human Evolution, Max Planck Institute for Evolutionary Anthropology, Leipzig, Germany; 2 Department of Palaeoanthropology and Messel Research, Senckenberg Research Institute, Frankfurt am Main, Germany; University of Utah, United States of America

## Abstract

Most of the morphological features recognized in hominin teeth, particularly the topography of the occlusal surface, are generally interpreted as an evolutionary functional adaptation for mechanical food processing. In this respect, we can also expect that the general architecture of a tooth reflects a response to withstand the high stresses produced during masticatory loadings. Here we use an engineering approach, finite element analysis (FEA), with an advanced loading concept derived from individual occlusal wear information to evaluate whether some dental traits usually found in hominin and extant great ape molars, such as the trigonid crest, the entoconid-hypoconulid crest and the protostylid have important biomechanical implications. For this purpose, FEA was applied to 3D digital models of three *Gorilla* *gorilla* lower second molars (M_2_) differing in wear stages. Our results show that in unworn and slightly worn M_2_s tensile stresses concentrate in the grooves of the occlusal surface. In such condition, the trigonid and the entoconid-hypoconulid crests act to reinforce the crown locally against stresses produced along the mesiodistal groove. Similarly, the protostylid is shaped like a buttress to suffer the high tensile stresses concentrated in the deep buccal groove. These dental traits are less functional in the worn M_2_, because tensile stresses decrease physiologically in the crown with progressing wear due to the enlargement of antagonistic contact areas and changes in loading direction from oblique to nearly parallel direction to the dental axis. This suggests that the wear process might have a crucial influence in the evolution and structural adaptation of molars enabling to endure bite stresses and reduce tooth failure throughout the lifetime of an individual.

## Introduction

Since decades scholars have focused their attention on the morphology of the occlusal surface of human and non-human primate teeth to gain insight on the food items each species is more adapted to process and to improve our understanding of early hominin diets and dietary adaptations [1–5]. Even though a certain amount of within-species variability in the food items consumed cannot be excluded, it is acknowledged that cusps with steeply inclined slopes are well suited to generate shear-cutting forces, suggesting a diet of both soft and ductile foodstuffs; conversely, rounded (or blunt) cusps are well suited for crushing, indicating a diet of hard, brittle foods [4,6,7].

However, during food processing teeth must solve another equally important function, namely they should be designed to resist failure while distributing forces produced during masticatory loading to their supporting structures [8–12]. These two main functions, food diminution and resilience to failure, act together in the occlusal part of the chewing cycle, the power stroke, during which food is comminuted and tooth-to-tooth contacts occur. Accordingly, beside the well-known variation in dental occlusal topography (i.e., steep or blunt cusps), which evolved to improve mechanical efficiency for food reduction, there should be other dental morphological features that simultaneously evolved to withstand occlusal loads.

Results from fracture mechanics suggest that, at least in great apes, dental material properties are less likely to be of concern than dental morphology (both internal and external architecture) in the load-bearing capacity of the teeth [13,14]. With regard to the internal architecture, the enamel thickness might be an example of such adaptation, as thick enamel allows both to increase wear resistance and to withstand and/or dissipate high masticatory loads [15–19]. Dental biomechanics suggest also that the arrangement of crystals within each enamel rod, enamel prism interweaving (decussation) and self-healing processes (growing fissures filled with organic fluids) are designed to better arrest crack growth in thick enamel driven by extended use or overloading [11,20–24].

Less attention, however, has been devoted to understand whether the external geometry of the teeth might optimize resilience on stress distribution. In an attempt to interpret the load-dissipation behavior of great ape molars, Macho and Spears [12] used two-dimensional (2D) finite element analysis (FEA) to suggest that modifications of the occlusal topography are more responsible for efficient load dissipation than increasing enamel thickness by 100%, which ultimately reduces maximum tensile stresses by only 15%. Magne and Belser [25] used the same approach (2D FEA) to evaluate the biomechanical behavior of opposing human molars in different load-cases. They observed that high stress levels were concentrated in the central groove of maxillary molars, and that enamel bridges and crests might reduce tensile stress locally, thus protecting crown biomechanics. Lucas and colleagues [17] suggested that the cingulum (a ridge encircling the base of a tooth) might be functional important to protect the neck of the tooth from margin cracks driven by tensile stresses. Indeed, margin cracks begin at the base of the enamel (at or near the cervix) and extend longitudinally toward the occlusal surface [7,15,26], and are a source of failure both in real and ceramic dental crowns [27]. Finally, Anderson and colleagues [28] used FEA in cone shape “teeth” to show that cingula structure might indeed be important to reduce tensile strains in the enamel.

Despite these works, little is known about the functional biomechanics of external dental features, mainly because all of the above studies have much simplified the complex three-dimensional (3D) geometry of the tooth either using sections (2D approach), or modeling the tooth as cone shape-like, or applying unrealistic occlusal loadings.

**Figure 1:**
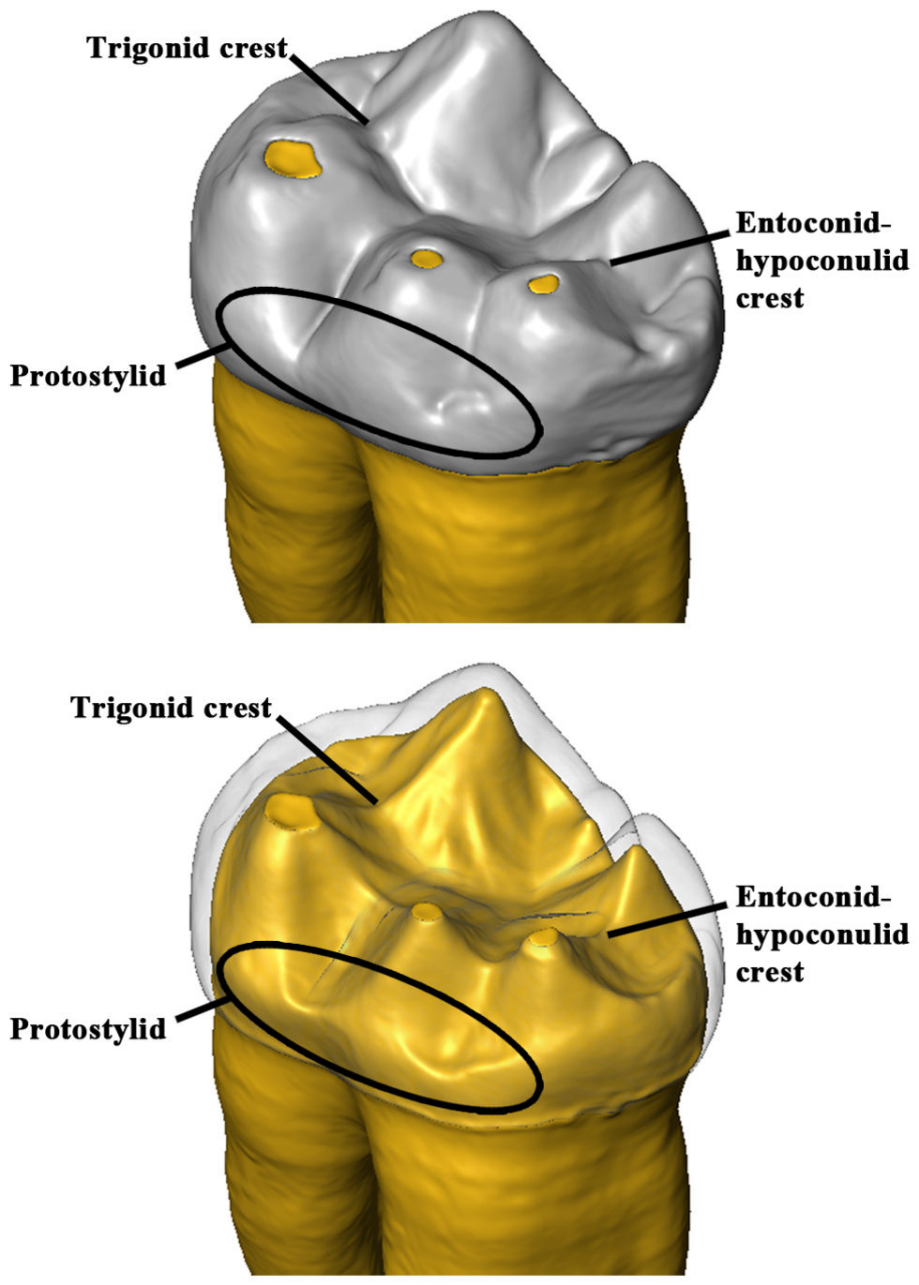
Digital reconstruction of the gorilla specimen ZMB-31626 (lower left second molar – LM_2_). The three dental traits examined in this study (protostylid, trigonid crest, entoconid-hypoconulid crest) are highlighted both in the crown (top) and in the enamel-dentine junction (bottom).

In this pilot study we used 3D FEA [29] with a newly developed advanced loading concept derived from individual occlusal wear information [8,30–32] to test whether some dental traits usually found in hominin and extant great ape lower molars, such as the trigonid crest pattern, the entoconid-hypoconulid crest and the protostylid (crest feature on the buccal wall of the crown, normally associated with the buccal groove [33,34]), might represent evolutionary responses to occlusal loadings (Figure 1). As African apes represent good models for understanding dental functional morphology in early hominins [35], we compared maximum principal stresses in 3D digital models of three *Gorilla* *gorilla* lower left second molars (LM_2_) during maximum intercuspation tooth-to-tooth contact, which might be more damaging to the tooth crown than food-tooth contacts because of increased localized stresses. The above mentioned dental traits (trigonid crest pattern, the entoconid-hypoconulid crest and the protostylid) are well expressed in gorilla molars, which are also characterized by tall cusps, long shearing crests and relatively thin enamel [36], presumably an adaptation to folivorous diet [37]. As our three gorilla LM _2_s differ in wear stages, we also aim to evaluate the effects of a reduced relief through wear on the stress distribution. Finally, a digital simulation was carried out to assess whether interrupting the continuity of the crests (the trigonid and the entoconid-hypoconulid crest, respectively) might affect the pattern of stress distribution.

## Materials and Methods

Three *Gorilla* *gorilla* female skulls from the Museum für Naturkunde, Humboldt Universität, Berlin, Germany were selected for 3D FEA. The three specimens (ID = ZMB-31435, ZMB-31626 and ZMB-83551, respectively) were selected both because of their complete dentition and because their LM_2_ differing in wear stage (after Smith [38]): ZMB-31435 = wear stage 1; ZMB-31626 = wear stage 3; ZMB-83551 = wear stage 4. We obtained permission from the Museum für Naturkunde (Humboldt Universität, Berlin) to micro-CT scan the skulls at the Bundesanstalt für Materialforschung und –prüfung, Berlin, Germany (scan parameters: 160kV, 150µA, 1.0mm copper filter, and 2400 views per rotation). Volume data were reconstructed using isometric voxels ranging between 61 and 65µm.

**Figure 2:**
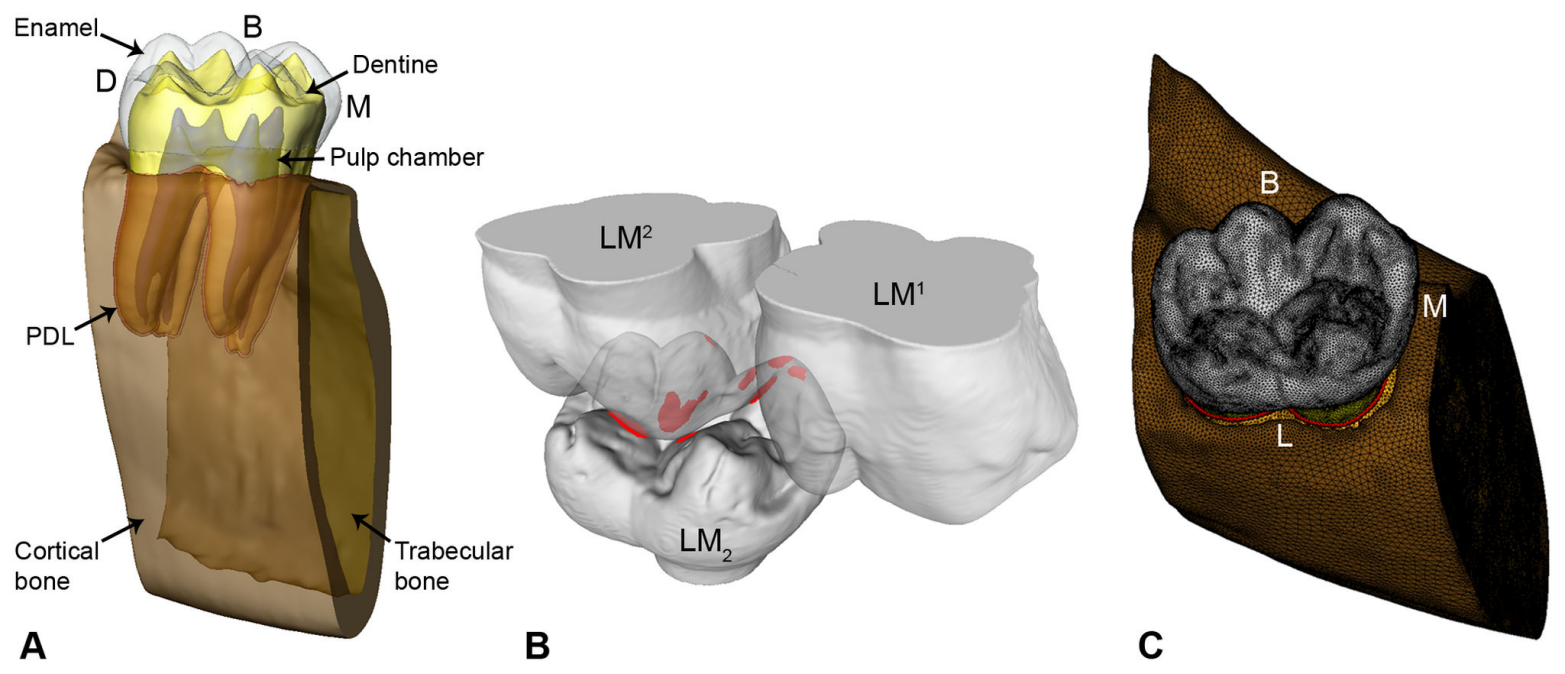
Basic steps to create the volumetric mesh and to recognize the contact areas for specimen ZMB-31435. A, dental tissues and supporting structures for the lower left second molar (LM_2_) of specimen ZMB-31435. B, collision detection for specimen ZMB-31435 in the Occlusal Fingerprint Analyser (OFA) software during maximum intercuspation contact situation; the LM^1^-LM^2^ are transparent to show the collision (red spots) in the occlusal surface of the LM_2_ (see also Video S1). C, the FE mesh of specimen ZMB-31435 consisting of 2,482,913 ten-nodded tetrahedral elements. PDL = periodontal ligament; B = buccal; D = distal; L = lingual; M = mesial.

To reduce the size of the digital models, we cut the mandibles distally to the socket of the lower left first molar (LM_1_) and mesially to the socket of the lower left third molar (LM_3_). Consequently, we considered only the bone tissues surrounding the LM_2_. Segmentation of the LM_2_ dental tissues (enamel, dentine and pulp chamber) and its supporting dental tissues (periodontal ligament - PDL, trabecular and cortical bone) was carried out in Avizo 7 (Visualization Sciences Group Inc.) (Figure 2A). For LM^1^-LM^2^, which were used to assess the occlusal contacts with LM_2_ (two-body interactions), only the external surface of the teeth was segmented. The final refinement of the digital models was carried out in Rapidform XOR2 (IN, US Technology, Inc., Seoul, Korea).

As described in previous contributions [8,30,32], the dental surface models of lower and upper molars were imported into the Occlusal Fingerprint Analyser (OFA) software to recognize the contact areas on the LM_2_ with the antagonistic teeth during the power stroke. The contact areas were automatically selected by the software, thus informing on the position where occlusal loads should be applied (red areas in Figure 2B see also Video S1-S3). In order to compare the pattern of stress distribution of the three gorilla specimens, the maximum intercuspation contact situation was selected. With regard to the loading direction, it has been already suggested, for maximum intercuspation, to apply perpendicular loads to the contact areas [8,30,39].

The surface models were then imported into HyperWorks Software (Altair Engineering, Inc.), where volumetric meshes (for enamel, dentine, pulp, PDL, cortical and trabecular bone shown in Figure 2C) were created using 10-nodes tetrahedral elements (Table S1). Information for material properties such as the elastic modulus -E, and the Poisson’s ratio were collected from the literature [13,40–43] and summarized in Table 1. All the biological materials represented in the models were considered homogeneous, linearly elastic and isotropic.

**Table 1:** Elastic properties of dental and bone tissues.

Materials	E^b^ (GPa)	Poisson’s ratio	References
Enamel	93	0.3	[13]
Dentin	18.6	0.31	[42]
Pulp	0.002	0.45	[43]
PDL^a^	0.0689	0.45	[41]
Alveolar bone	11.5	0.3	[40]
Cortical bone	13.7	0.3	[42]

^a^ Periodontal ligament; ^b^ elastic modulus

**Figure 3:**
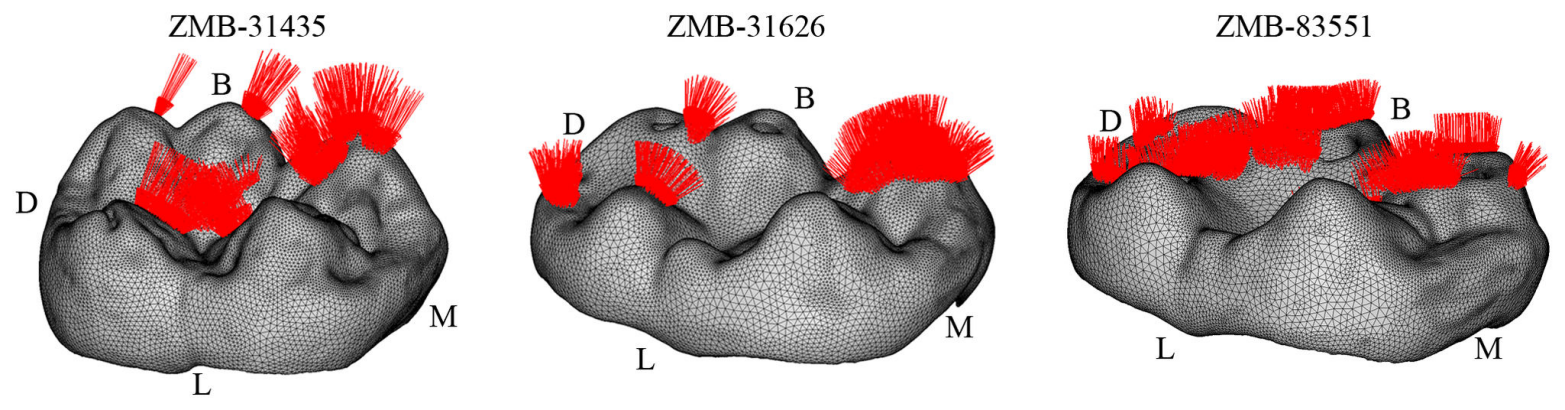
Loading position and direction for specimen ZMB-31435, ZMB-31626 and ZMB-83551. For each lower left second molar (LM_2_) only the volumetric mesh of the enamel is displayed. The load (red arrows) was distributed proportionally according to the occlusal contact areas detected in the Occlusal Fingerprint Analyser (OFA) software (see also Video S1-S3). B = buccak; D = distal; L = lingual; M = mesial.

Boundary constraints were applied to the medial and distal cut surfaces of the mandible section following indications provided by Benazzi et al. [8]. The load (uniform pressure) was distributed proportionally according to the occlusal contact areas detected in the OFA software (Figure 3) and was such that the magnitude of the resultant vector was equal to 150N. Since the three LM_2_ specimens have similar size (mesiodistal diameter = 17.8 ± 0.3; buccolingual diameter = 15.1±0.3), scaling of the volumetric meshes was not required.

The stress state patterns were qualitatively and quantitatively compared according to the first maximum principal stresses criterion for brittle materials [8,9,30,32,44,45], wherein the stresses inform about tensile behaviour in specific sites of the volumetric meshes.

**Figure 4:**
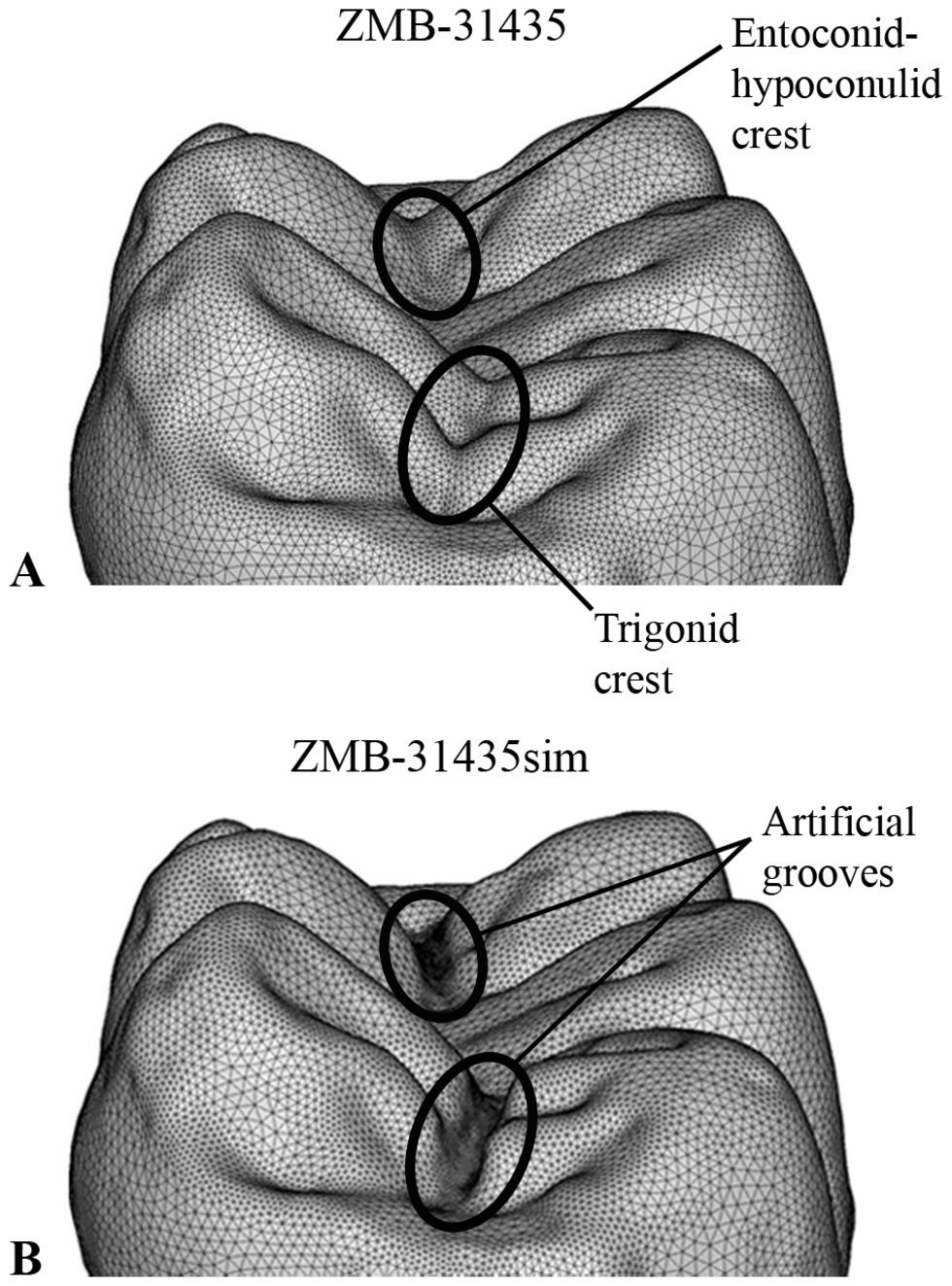
The enamel volumetric meshes of specimen ZMB-31435 and ZMB-31435sim. A, the volumetric mesh of specimen ZMB-31435 LM_2_ with highlighted the crests considered in the simulation. B, the volumetric mesh with artificial mesiodistal grooves interrupting the trigonid and entoconid-hypoconulid crests (specimen ZMB-31435sim).

Finally, the volumetric mesh of ZMB-31435 LM_2_ was digitally modified in HyperWorks software to study the relationships between the presence/absence of dental traits and the pattern of stress distribution. In the simulation (hereafter referred as ZMB-31435sim), the midtrigonid and entoconid-hypoconulid crests (Figures 1, 4A) were crossed by mesiodistally directed grooves (Figure 4B), which connect the central fossa to the anterior and posterior fovea respectively, thus interrupting the continuity of the crests. With regard to the enamel thickness in the artificial grooves, we averaged the values measured in the central fossa and in both the fovea (~0.77mm). In order to better evaluate the effects of these morphological changes on the pattern of stress distribution, new loading conditions were applied based on a suitable time-step of phase I (Video S1). As described by Benazzi and colleagues [30,32] about loading direction during phase I, we computed the resultant force from the normal force to the contact area and the tangential force, the latter given by the coefficient of friction times of the normal force. We used a coefficient of friction of 0.2, which was found for wet conditions [46].

## Results

**Figure 5:**
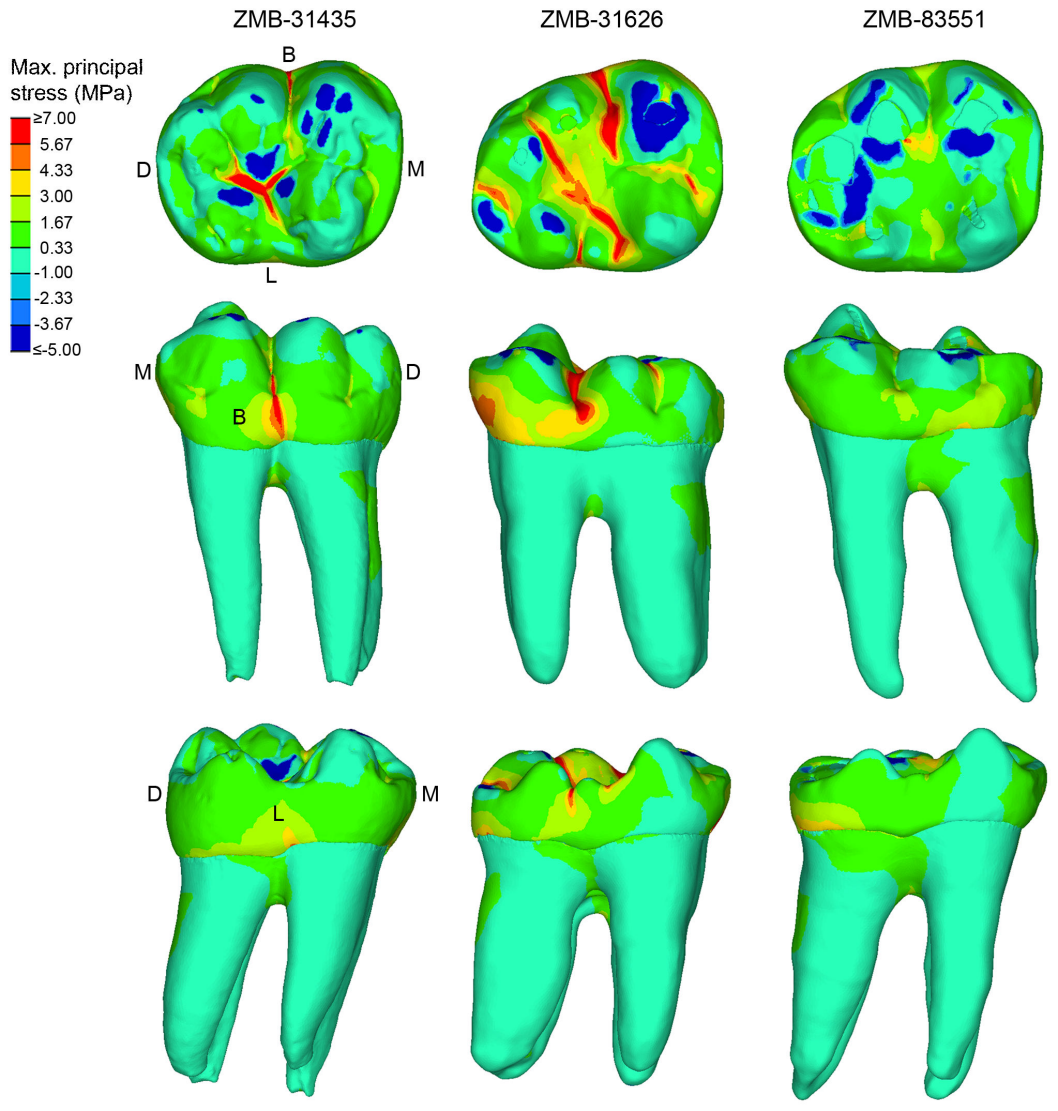
Maximum principal stress distribution observed in ZMB-31435 LM_2_ (left), ZMB-31626 LM_2_ (middle) and ZMB-83551 LM_2_ (right) during maximum intercuspation contact. Blue areas mark the position were occlusal forces were applied. First row = occlusal view; second row = buccal view; third row = lingual view. B = buccal; D = distal; L = lingual; M = mesial.

**Figure 6:**
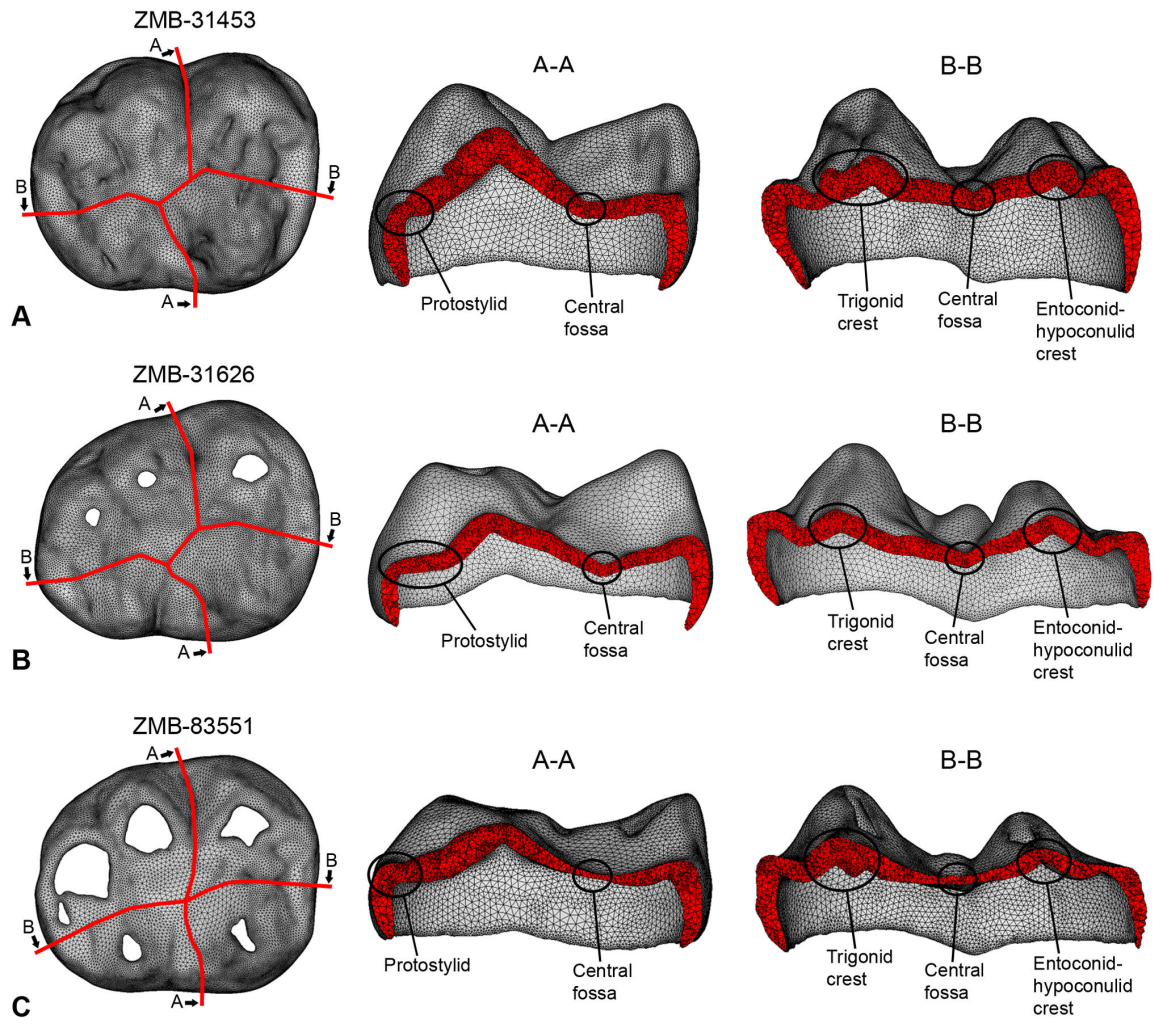
Sections of the enamel volumetric meshes along the buccolingual groove (A-A) and mesiodistal groove (B–B). A, specimen ZMB-31435 LM_2_. B, specimen ZMB-31626 LM_2_. C, specimen ZMB-83551 LM_2_.

The distribution of maximum principal stress during maximum intercuspation contact for the three gorilla specimens is shown in Figure 5. In specimen ZMB-31435 LM_2_ (wear stage 1) and specimen ZMB-31626 LM_2_ (wear stage 3) tensile stresses are observed in the grooves of the occlusal surface. Sections of the enamel along the buccolingual and mesiodistal grooves (Figure 6A,B; section A-A and B–B, respectively), indicate that regions of the grooves characterized by relative thin enamel, such as the central fossa (ZMB-31435LM_2_ = ~0.79mm; ZMB-31626LM_2_= ~0.63mm), usually concentrate tensile stresses. Both specimens also experience tensile stresses in the groove between the two main buccal cusps (protoconid and hypoconid). However, in ZMB-31626 LM_2_ tensile stresses interest the entire buccal groove, ending at the level of the protostylid, which is well-developed and shaped like a shelf (Figure 6B). Conversely, in ZMB-31435 LM_2_ tensile stresses are concentrated in the cervical end of the buccal groove and ultimately reach the cervix. This region of the tooth is characterized by a poorly expressed protostylid and relatively thinner enamel, at least when compared with the occlusal half of the buccal groove (Figure 6A).

In ZMB-31626 LM_2_ tensile stresses involve also the buccodistal groove (between hypoconid and hypoconulid cusps), the lingual groove, which bifurcates in correspondence of an accessory cusp (cusp 7), as well as the anterior and posterior fovea. The propagation of the tensile stresses along the mesiodistal groove is interrupted by the trigonid and entoconid-hypoconulid crests, which are particularly massive in ZMB-31435 LM_2_ (Figures 5, 6A,B). In both specimens the crests present an increase in enamel thickness compared to the thickness measured in both the central fossa and in the anterior/posterior fovea (i.e., in ZMB-31435 LM_2_: trigonid crest = ~1.49mm; entoconid-hypoconulid crest = ~1.15mm; anterior fovea = ~0.77mm; posterior fovea = ~0.78mm).

In the worn specimen ZMB-83551 LM_2_ (wear stage 4) the force was applied on a larger portion of the occlusal surface when compared with ZMB-31626 LM_2_ and particularly with ZMB-31435 LM_2,_ encompassing almost all the cusps. The tooth is basically subjected to compressive loads resulting in compressive contact stresses. Tensile stresses of low magnitude are only observed between the protoconid and hypoconid cusps, but they do not interest the buccal groove (Figure 5). Despite tooth wear has notably reduced the enamel thickness (Figure 6C), tensile stresses do interest neither the mesiodistal groove nor the central fossa.

**Figure 7:**
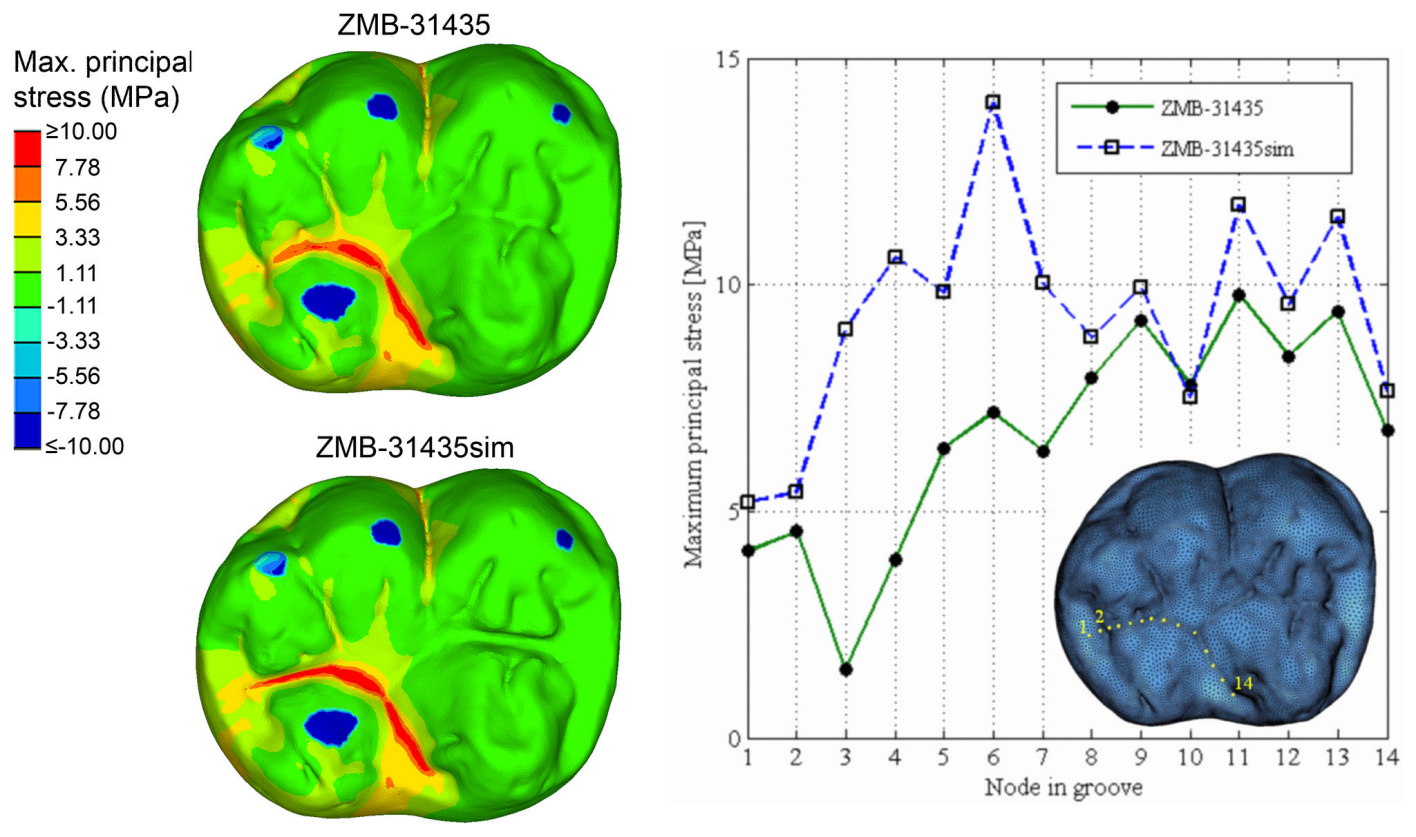
Maximum principal stress distribution in ZMB-31435 LM_2_ and ZMB-31435sim LM_2_ during a representative time-step of phase I. Blue areas on the occlusal surface mark the position were occlusal forces were applied, and red areas show maximum tensile stress. The plot on the right side shows the differences in tensile stress values between the two specimens based on 14 homologous nodes selected on the occlusal grooves.

To compare specimen ZMB-31435 and ZMB-31435sim (Figure 4A and 4B, respectively) we used a representative time-step of phase I with most of the cusps in occlusal contacts with the antagonistic teeth. Forces were applied on the buccal cusps (protoconid, hypoconid and hypoconulid) and on the entoconid cusp, whereas no contact was detected on the metaconid cusp by the OFA software. Based on this loading condition, an increase in tensile stress values is observed in the artificial groove between the entoconid and hypoconulid cusps, but no changes in stress distribution are observed in the artificial groove between protoconid and metaconid cusps (Figure 7).

## Discussion and Conclusions

In the last years, several scholars have warned about the importance of crown geometry and loading directions on the distribution of stresses developing within a tooth during occlusal loadings [12,28,47]. Knowledge of these effects has obvious implications not only for the understanding of the relation between functional biomechanics and tooth morphology in an adaptive and evolutionary context [12], but also for modern dentistry to appropriately design the architecture and shape of artificial crowns and to test materials for tooth restoration [14].

The patterns of stress distribution derived from the advanced FEA in the three gorilla specimens, considering both the occlusal loading position/direction and the specific occlusal morphology, informs about differences in reaction force depending on the architecture of the crowns and compressive antagonistic contacts.

Our results confirm preliminary suggestions that grooves and fissures on the occlusal surface concentrate high tensile stresses [25,30,48], particularly evident in unworn up to moderately worn teeth (specimen ZMB-31435 and ZMB-31626, respectively), wherein occlusal loads are transferred to the crown following a direction dictated by the cusp inclination. As cusps differently incline, the occlusal force will be fragmented in several lateral loadings that ultimately create tensile stresses in the concave regions between the cusps (Figures 5, 7). With regard to ZMB-31626 LM_2_, forces applied on the lingual slope of the protoconid and in the talonid (namely, in the entoconid, hypoconulid and distal marginal ridge) bend the crown mainly about the buccolingual axis, inducing tensile stresses along the buccolingual groove. In such condition, the protostylid concentrates high tensile stresses and reduces tensile stresses at the thin-enamelled cervix, and thus it might protect the tooth from marginal cracking as previously suggested [17,28]. Despite its relatively thin enamel (relatively to the average thickness of the groove), the shelf-like morphology of the ZMB-31626 LM_2_ protostylid fragments the tensile stresses in the double fold structure. The discontinuity of the slope of the buccal groove creates an irregular change in geometry, making the tensile stress locally concentrated around the irregularly geometrical area, which is relatively far away from the cervix. Specimen ZMB-31435 LM_2_ confirms that the protostylid is a dental feature subjected to tensile stresses, but it also emphasizes that poorly expressed protostylids are less suitable to reduce tensile stresses at the cervix. Most of the tensile stresses observed in ZMB-31435 LM_2_ during maximum intercuspation are concentrated in the central fossa (Figure 5), and we interpret that the trigonid crest and the entoconid-hypoconulid crest reinforce the crown against stresses produced along the mesiodistal groove. This is also evident in the slightly more worn specimen ZMB-31626 LM_2_, wherein tensile stresses occur not only in the buccolingual grooves, but also in the anterior and posterior fovea, hence beside the two crests. Results of the simulation during phase I shown in Figure 7 underline our assumptions, but they also emphasize that presence/absence of a crest has only local effects, as previously suggested by Magne and Belser [25]. The occlusal load applied to ZMB-31435sim LM_2,_ without trigonid and entoconid-hypoconulid crests, was merely concentrated in the talonid region, with obvious increase in the tensile stress values on the simulated distal groove, but without any effect on the simulated mesial groove. Therefore, it is conceivable that several dental features work in concert to reduce locally dangerous effects of high tensile stresses. In some cases, as suggested by Macho and Spears [12] and as observed in the protostylid of specimen ZMB-31626 LM_2_, the presence and its characteristic expression may be responsible to withstand stress distribution. However, in other circumstances the interaction between the external topography and the internal architecture, i.e., the thickened enamel in the crest, probably plays an important role (Figure 6). Similar roles can be supposed also for other occlusal features along the border of the crown, such as the mesial and distal marginal ridges and the lingual crest (the crest between the lingual cusps, metaconid and entoconid). Much more works are needed to explore the biomechanical effects of dental features, and the correspondence between external and internal architecture and its functional implications.

In worn teeth, dental features such as the trigonid crest and the entoconid-hypoconulid crest are not exempted from tooth wear. However, the progressive deterioration of these features occurs in concert with morphological alterations of the entire occlusal surface, which ultimately reduces locally directed stresses and improves the dispersion of occlusal forces. Indeed, the buccal cusps of the worn ZMB-83551 LM_2_ specimen are lower and flatter than in ZMB-31626 LM_2_ and particularly in ZMB-31435 LM_2_, so that the load directions change from oblique to nearly parallel direction to the dental axis (Figure 3). Moreover, as the contact areas increased in number and extension, the occlusal force per unit of surface area decreases. As a result, the tensile stresses in the crown decrease meaningfully. Similar conclusions have been emphasized in restorative dentistry, where it has been observed that reduction of cusp height reduces tensile stress values (i.e., [49]). It is also worthwhile to note that the low tensile stresses observed in the occlusal surface of ZMB-83551 LM_2_, wherein tooth wear has notably reduced the enamel thickness and has partially removed and flattened both the occlusal grooves and the central fossa (Figure 6C), further suggest that the occlusal topography might be more important for efficient load dissipation than the enamel thickness, supporting previous assumptions by Macho and Spears [12]. To summarize, we do not suggest that it is better to have a completely worn and flat occlusal surface, because there is no doubt that occlusal reliefs are important for food processing [6]. We observe, however, that a reduction in cusp’s steepness due to tooth wear reduces tensile stresses in the crown, and this decrease might be useful when morphological features such us, i.e., crests and ridges, grooves, enamel thickness, crown height, have been either completely removed or heavily reduced by tooth wear. We suggest that a strong interaction subsists among dental morphology, occlusal load and tooth wear. Some dental features might be useful to suffer high tensile stresses in unworn or moderately worn teeth, wherein the occlusal load is applied in relatively small contact areas along cusp slopes, generating non-axial loadings. However, a decrease in tensile stresses due to tooth wear makes buttresses-like features less important to compensate loads.

Therefore, as suggested in a recent contribution [50], the wear process with its loss of dental tissue and the reduction of the occlusal relief might have had a crucial influence in the evolutionary adaptation of teeth, augmenting to endure specific stresses in advanced periods of an individual’s lifetime. It seems that we observe an evolutionary compromise, and tooth evolution and dental biomechanics can only be understood, if we further investigate tooth function in respect to the dynamic changes of tooth structures during the lifespan of individuals.

Maybe in other extant and extinct hominoid primates, dental features such as the protostylid, the trigonid and the entoconid-hypoconulid crests represent plesiomorphic traits that do not provide any functional advantage for the tooth, while other features not considered in this study (e.g., crenulated occlusal surface, complexity of occlusal grooves pattern, bulging of cusp’s buccal wall, or the Carabelli cusp and oblique crest in the upper molar) might be also important to reinforce the crown, limiting dangerous effect of high tensile stresses due to occlusal loadings.

Finally, it is important to underline some limits of our FE analysis that should be addressed in future works. We have attributed isotropic property to the enamel, but it has been suggested that enamel should be considered anisotropic [24]. We have investigated only a static occlusal loading condition. Even though we are confident about our results (we have chosen the most critical scenarios with maximum individual tooth-tooth contacts), further works should also consider kinetic loading conditions during tooth-food-tooth contacts, which may provide a more realistic picture of the stress distribution in the tooth. Moreover, we have considered only three specimens due to the efforts required to develop the FE models. Even though we do believe these specimens are morphologically representative, we emphasize that the results described from this pilot work should be extended considering not only other extant hominoid species, but also hominin fossil species. It is well known that fossil African hominin taxa such as australopiths differ from extant African apes in having thicker enamel and generally lower and blunt molar reliefs [4,51], suggesting that less tensile stresses occur in maximum intercuspation. Since early *Homo* specimens show intermediate occlusal reliefs and surface sloping between *Gorilla* and *Pan* [4], we expect also an intermediate tensile stress distribution, depending on the expression of edges and grooves on their occlusal surfaces. However, even if we assume that such features (as well as other morphological traits such as accessory cusps, complex groove/fissure patterns, general crown height and flaring, cusp size proportion) contribute to withstand occlusal loads, more investigations are required for a better understanding of the biomechanical behavior and the evolution of hominin dental features.

## Supporting Information

Table S1 Numbers of nodes and tetrahedral elements for each specimen. (DOC)

Video S1 Simulation of the individual occlusal “power stroke” of specimen ZMB-31435 applying the Occlusal Fingerprint Analyser (OFA) software. The OFA calculates a relief-guided pathway of antagonistic tooth rows from collision detection, deflection and break-free algorithms for user-defined timesteps. The contact areas of maximum intercuspation have been chosen for applying loads in the FE models.
(MP4)

Video S2 Simulation of the individual occlusal “power stroke” of specimen ZMB-31626 applying the Occlusal Fingerprint Analyser (OFA) software. The OFA calculates a relief-guided pathway of antagonistic tooth rows from collision detection, deflection and break-free algorithms for user-defined timesteps. The contact areas of maximum intercuspation have been chosen for applying loads in the FE models.
(MP4)

Video S3 Simulation of the individual occlusal “power stroke” of specimen ZMB-83551 applying the Occlusal Fingerprint Analyser (OFA) software. The OFA calculates a relief-guided pathway of antagonistic tooth rows from collision detection, deflection and break-free algorithms for user-defined timesteps. The contact areas of maximum intercuspation have been chosen for applying loads in the FE models.
(MP4)
