# Supplementary material for: Unravelling the Functional Biomechanics of Dental Features and Tooth Wear
Source: PLoS One. 2013 Jul 23;8(7):e69990. doi: 10.1371/journal.pone.0069990 (PMC3720920; doi:10.1371/journal.pone.0069990)
Supplement: Table S1 — (DOC) [file pone.0069990.s001.doc]

| **Table S1.** Numbers of nodes and tetrahedral elements for each specimen | | |
| --- | --- | --- |
|
| Specimen | Nodes | Tetrahedral elements |
| ZMB-31435 | 3,455,306 | 2,482,913 |
| ZMB-31626 | 3,387,544 | 2,455,178 |
| ZMB-83551 | 3,058,453 | 2,211,084 |
| ZMB-31435sim^a^ | 3,498,887 | 2,511,939 |
| ^a^Digital simulation of mesiodistal grooves | | |
